# *Leishmania mexicana*: Novel Insights of Immune Modulation through Amastigote Exosomes

**DOI:** 10.1155/2020/8894549

**Published:** 2020-12-02

**Authors:** Laura Enedina Soto-Serna, Mariana Diupotex, Jaime Zamora-Chimal, Adriana Ruiz-Remigio, José Delgado-Domínguez, Rocely Buenaventura Cervantes-Sarabia, Adriana Méndez-Bernal, Alma Reyna Escalona-Montaño, María Magdalena Aguirre-García, Ingeborg Becker

**Affiliations:** ^1^Unidad de Investigación en Medicina Experimental, Facultad de Medicina, Universidad Nacional Autónoma de México, Hospital General de México, Dr. Balmis 148, Col. Doctores, CP 06726 Ciudad de México, Mexico; ^2^Facultad de Medicina Veterinaria y Zootecnia, Departamento de Patología y Microscopía electrónica, Universidad Nacional Autónoma de México, Circuito Exterior, Ciudad Universitaria, Av. Universidad 3000, CP 04510 Ciudad de México, Mexico; ^3^Facultad de Medicina, División de Investigación, Unidad de Investigación UNAM-INC (Instituto Nacional de Cardiología Ignacio Chávez), Juan Badiano No. 1, Col. Sección XVI, 14080 Ciudad de México, Mexico

## Abstract

Exosomes are extracellular microvesicles of endosomal origin (multivesicular bodies, MVBs) constitutively released by eukaryotic cells by fusion of MVBs to the plasma membrane. The exosomes from *Leishmania* parasites contain an array of parasite molecules such as virulence factors and survival messengers, capable of modulating the host immune response and thereby favoring the infection of the host. We here show that exosomes of *L. mexicana* amastigotes (aExo) contain the virulence proteins gp63 and PP2C. The incubation of aExo with bone marrow-derived macrophages (BMMs) infected with *L. mexicana* led to their internalization and were found to colocalize with the cellular tetraspanin CD63. Furthermore, aExo inhibited nitric oxide production of infected BMMs, permitting enhanced intracellular parasite survival. Expressions of antigen-presenting (major histocompatibility complex class I, MHC-I, and CD1d) and costimulatory (CD86 and PD-L1) molecules were modulated in a dose-dependent fashion. Whereas MHC-I, CD86 and PD-L1 expressions were diminished by exosomes, CD1d was enhanced. We conclude that aExo of *L. mexicana* are capable of decreasing microbicidal mechanisms of infected macrophages by inhibiting nitric oxide production, thereby enabling parasite survival. They also hamper the cellular immune response by diminishing MHC-I and CD86 on an important antigen-presenting cell, which potentially interferes with CD8 T cell activation. The enhanced CD1d expression in combination with reduction of PD-L1 on BMMs point to a potential shift of the activation route towards lipid presentations, yet the effectivity of this immune activation is not evident, since in the absence of costimulatory molecules, cellular anergy and tolerance would be expected.

## 1. Introduction

Leishmaniasis is a zoonotic disease caused by different species of the genus *Leishmania*, an intracellular parasite that infects the mammalian host after being transmitted by the sand fly vector (*Phlebotomus* spp. and *Lutzomyia* spp.) during its bloodmeal [1]. *Leishmania* promastigotes are phagocytosed by antigen-presenting cells (APCs), such as macrophages and dendritic cells, where they differentiate into amastigotes and begin their replication [2]. Macrophages play multiple functions during the infection, including parasite elimination, antigen-presentation for T cell activation, and cytokine production [3]. These coordinated response mechanisms are necessary to limit disease progression [3]. The parasite has developed diverse evasion strategies for its survival within the mammalian host, including inhibition of the respiratory burst, thereby lowering the toxic reactive oxygen intermediates (ROI) and reducing nitric oxide (NO) production, both of which are important leishmanicidal mechanisms [3, 4]. NO production is regulated by the inducible nitic-oxide synthase (iNOS) through activation of inducible nuclear factors, including nuclear factor kappa-light-chain enhancer of activated B cell (NF-*κ*B) [5]. Parasite evasion mechanisms are mediated by virulence factors as a glycoprotein of 63 kDa (gp63) and the elongation factor 1-*α* (EF-1*α*), which activates SHP-1 (PTP) in macrophages, thus avoiding signaling through STAT-1 and inhibiting transcription factors such as NF-*κ*B and AP-1 [6–9]. Furthermore, the protein phosphatase 2C (PP2C), found in the secretome of various *Leishmania* species, regulates proinflammatory cytokine production in macrophages [10]. This protein has been implicated in pathogenic mechanisms favoring infectivity of various parasites such as *Plasmodium falciparum* and *Toxoplasma gondii* [11, 12].

Recently, exosomes secreted by either *Leishmania* or infected macrophages have been proposed as one of parasite evasion strategies within host cells, since they are able to modulate effector mechanisms and the expression of surface molecules involved in antigen presentation and costimulation [13, 14]. Exosomes are extracellular nanovesicles measuring 30-150 nm [15–17]. They are formed within multivesicular bodies (MVBs) and contain varied amounts of nucleic acids, proteins and lipids, which are released to extracellular spaces by almost all cells, serving as intercellular communication systems of eukaryotes and prokaryotes [16–18]. Exosomes regulate central biological processes, such as immune responses, and are found in diverse body fluids such as urine, saliva, blood and serum through which they can be distributed throughout the body [16].

In leishmaniasis, parasite-derived exosomes have been shown to contain the glycoprotein gp63 (a *Leishmania* zinc-metalloprotease abundantly expressed on the parasite surface), which can influence host signaling mechanisms [19, 20]. Parasite exosomes have also been shown to contain heat-shock proteins (HSP) 70 and 100 which aid parasite progression in the mammalian host [19]. Another protein recently described in *L. major* exosomes is the phosphatase LmPRL, which favors parasite growth and differentiation within macrophages, and exosomes of *L. infantum* interfere with activation of APCs by lowering their costimulatory molecules CD86 and CD40 [21, 22]. Yet exosomes of *L. mexicana* parasites that are capable of producing severe progressive cutaneous diseases remain to be explored. Therefore, in the current study, we analyzed the immunomodulatory effect exerted by exosomes of *L. mexicana* amastigotes on infected bone marrow-derived macrophages (BMMs).

## 2. Material and Methods

### 2.1. Animals

BALB/c mice were bred and housed at the animal facilities at the Unit of Experimental Medicine Research of the Medical School-UNAM, following the National Ethical Guidelines for animal health NOM-062-ZOO-1999 and the guidelines recommended for animal care by the ethical committee of the Medical School-UNAM.

### 2.2. Parasite Culture

*Leishmania mexicana* (MHOM/MX/2011/Lacandona) amastigotes were aspirated from footpad lesions of infected BALB/c mice and cultured at 33°C in Grace's insect medium, pH 5.5 (Gibco, Invitrogen Corporation, Gran Island, NY, USA), supplemented with 20% of heat-inactivated fetal bovine serum (FBS) (Biowest, Riverside MO, USA), 2 mM L-glutamine, 0.35 g/L sodium bicarbonate, and 1% Pen/Strep (100 IU/mL-100 *μ*g/mL) (Sigma-Aldrich, St. Louis, MO, USA), according to procedures previously described with some modifications [23].

### 2.3. Depletion of FBS-Derived Exosomes

Exosomes naturally present in FBS were depleted as previously described with some modifications [24]. Briefly, the serum was serially centrifuged at 300 x *g*/10 min, 1000 x *g*/20 min, and 3000 x *g*/30 min. Thereafter, it was filtered with a 0.22 *μ*m filter unit (Millex GV, Merck Millipore, Bedford, MA, USA) and ultracentrifuged at 100,000 x *g* during 90 min. The elimination of microvesicles was evidenced by flow cytometry (FACS Aria BD, San Jose, CA, USA), using reference beads measuring 0.1–2 *μ*m (Size reference kit, Invitrogen, Carlsbad, CA, USA) (Supplementary 1).

### 2.4. Growth Kinetics of *L. mexicana* Amastigotes

*L. mexicana* amastigotes were cultured for 10 days in modified complete Grace's insect medium supplemented with either 20% normal FBS or 20% exosome-free FBS (FBS/-exo). Amastigote cultures were initiated at 2 × 10^6^ parasites/mL from stock cultures in their exponential growth phase. For counting, the parasites were passed through 27 G syringe needles three to five times to disrupt aggregates of amastigotes and were fixed in 2% glutaraldehyde for daily count in a Neubauer chamber. Amastigote viability was verified with 0.2% erythrosin B (Sigma-Aldrich).

### 2.5. Isolation of *L. mexicana* Amastigote Exosomes (aExo)

*L. mexicana* exosomes were isolated from supernatants of amastigotes grown in axenic cultures in Grace's modified medium containing 20% FBS/-exo until reaching their stationary phase (7 days). Exosomes were enriched by differential centrifugation according to the protocol previously described [25]; thereafter, they were isolated with an ExoEasy Maxi kit (Qiagen, Hilden, Germany). Briefly, the parasites were centrifuged at 3,202 x *g* for 30 min, and the collected supernatant was filtered through a 0.22 *μ*m filter unit (Filter systems, Corning, Tewksbury, MA, USA). The supernatant was transferred to 26.3 mL polycarbonate tubes (Beckman Coulter, Fullerton, CA, USA) and ultracentrifuged at 100,000 x *g* for 1 h at 4°C. The pellet was suspended in 200 *μ*l phosphate-buffered saline, pH 7.2 (PBS) (Dulbecco, Caisson, CA, USA), and aExo were isolated with an ExoEasy Maxi kit, according to the manufacturer's instructions. Isolated exosomes were stored at -70°C until use.

### 2.6. Transmission Electron Microscopy (TEM)

For TEM visualization, aExo were fixed in 2.5% glutaraldehyde in 0.1 M sodium cacodylate buffer and mounted in formvar coated carbon-nickel grids. Uranyl acetate (2%) was used as a negative stain reagent. Samples were analyzed in a transmission electron microscope (JEOL 1010, Tokyo, Japan).

### 2.7. Protein Extraction

Isolated exosomes and stationary phase amastigotes were lysed in RIPA buffer (10 mM TrisHCl pH 7.4, 150 mM NaCl, 1 mM EDTA, 1% NP-40) in a cocktail of protease and phosphatase inhibitors (Sigma-Aldrich) and sonicated three times (0.5 s pulses/32% amplitude) at 22°C (SONICS-Vibra cell, Newtown, CT, USA). Proteins were quantified using an EZQ fluorescence kit (Invitrogen Molecular Probes, Eugene, OR, USA), according to the manufacturer's instructions. Protein integrity was verified by silver staining (Biorad, Hercules, CA, USA).

### 2.8. SDS-PAGE and Western Blot

Protein profiles of gp63, HSP70, PP2C, CD63, and CD9 from amastigotes or exosomal lysates (5 *μ*g) were analyzed by 10% SDS-PAGE in Tris-glycine-SDS (25 mM Tris, 0.1% SDS). For immunoblotting, proteins were transferred onto immobilon-P membranes (Millipore, Billerica, MA, USA). Thereafter, membranes were washed and blocked with 5% nonfat milk (Biorad) in TBS-T 1x (20 mM Tris-HCl, 150 mM NaCl, 0.005% Tween 20) for 1 h. As positive controls, gp63 (purified from *L. mexicana*), HSP70 (a protein lysate of cell-line 563), PP2C (recombinant PP2C of *L. major*), CD63 (protein lysate of the THP-1 cell line), and CD9 (protein lysate of ovarian cell line) were used (1 *μ*g of each protein). The blots were incubated in TBS-T/milk overnight at 4°C with rabbit polyclonal antibodies: anti-gp63 and anti-PP2C (both generated in our laboratory against native gp63 from *L. mexicana* promastigotes and recombinant *L. major* PP2C), both diluted 1 : 1000; mouse monoclonal antibodies: anti-HSP70 (Biolegend, San Diego, CA, USA), anti-CD63, and anti-CD9 (Abcam, Cambridge, MA, USA) (all at 1 : 5000 dilution). Thereafter, the membranes were washed with TBS-T for 1 h and incubated with secondary antibodies HRP-conjugated goat anti-rabbit IgG or HRP-conjugated goat anti-mouse IgG, respectively, at a 1 : 5000 dilution (Biomeda, Foster City, CA, USA) in TBS-T 1X with 1% nonfat milk. Finally, the membranes were washed ten times in TBS-T, and the bands were visualized by chemiluminescence substrate (Luminata Forte Western HRP Substrate, Millipore, USA), according to the manufacturer's instructions.

### 2.9. Bone Marrow-Derived Macrophages (BMMs)

BMMs were differentiated from bone marrow stem cells obtained from the femurs and tibias of BALB/c mice. Briefly, long bones of BALB/c mice were aseptically removed, and cells were flushed out with ice-cold PBS. The cells (2 × 10^6^) were transferred in Petri dishes (Falcon, Corning, New York, NY, USA) in RPMI-1640 medium (Thermo Fisher Scientific) supplemented with 20% FBS and 20% L929 fibroblast culture supernatants, as a source of macrophage-stimulating factor (M-SCF) [26]. They were incubated at 37°C in 5% CO_2_ for 7 days. Adherent BMMs were collected, and purity was analyzed by flow cytometry (FACS Canto II, BD, Becton Dickson, San José, CA, USA). Staining with mouse monoclonal antibody (mAb) anti-F4/80 (FITC) (Biolegend) showed that 98% of the cells were macrophages.

### 2.10. Confocal Microscopy

To visualize the internalization of parasite exosomes by BMMs, green fluorescent exosomes were obtained from *L. mexicana* parasites transfected with superfolder variant of the green fluorescent protein (sfGFP) according to the previously reported method [27]. Exosomes of *L. mexicana*^sfGFP^ were isolated from amastigotes and promastigotes and comparatively analyzed for their intracellular green fluorescence intensity. For confocal microscopy, exosomes of *L. mexicana*^sfGFP^ promastigotes were used, since these showed a stronger intracellular fluorescent signal (data not shown). BMMs (2.5 × 10^5^) were incubated with exosomes (10 *μ*g/mL) in 24-well culture tissue plates containing glass coverslips in RPMI-1640 supplemented with 10% FBS/-exo for 2 or 6 h at 37°C and 5% CO_2_. As control, nonstimulated cells were used. BMMs attached to coverslips were washed with PBS and intracellular staining was done with mouse anti-CD63 (PE) (Biolegend). Briefly, BMMs were fixed with 2% paraformaldehyde (PFA) for 20 min at 4°C and washed with 1x Perm/Wash buffer (Biolegend). Staining was done with anti-CD63 diluted 1 : 100 in permeabilization solution for 30 min at 4°C. Thereafter, cells were washed again with 1x Perm/Wash and nuclei were counterstained with DAPI in fluoroshield mounting medium (Sigma-Aldrich). Stimulated BMMs were visualized by confocal microscopy (Leica, TCS SP5, Leica Microsystems, USA), using the software LAS X v. 3 to image analysis.

### 2.11. Infection of BMMs (iBMMs)

BMMs (3 × 10^5^) were plated in 96-well tissue culture plates (Falcon-Corning) in complete RPMI-1640 medium supplemented with 10% of FBS/-exo and centrifuged at 300 x *g* for 5 min to allowed adherence. Cells were incubated with *L. mexicana* amastigotes at 1 : 5 (cell/amastigotes) ratio, and to synchronize the infection, the plates were centrifuged at 2,450 x *g* for 5 min. Then, the plates were incubated for 2 h at 33*°*C, an ideal condition for infection of BMMs by amastigotes [28], and thereafter, they were cultured at 37*°*C with 5% CO_2_ (to optimize culture conditions for BMMs) for 24 h.

### 2.12. Nitric Oxide (NO) Production

NO production by iBMMs stimulated with aExo was determined by measuring the accumulated levels of nitrites in the cell culture media [29]. Briefly, iBMMs were stimulated with LPS (100 ng/mL) and different concentrations of aExo in suspension (2.5, 5, 10, 25 and 50 *μ*g/mL) for 24 h in RPMI-1640 medium supplemented with 10% FBS/-exo at 37*°*C with 5% CO_2_. As controls, nonstimulated iBMMs and iBMMs stimulated only with LPS (100 ng/mL) were used. The supernatants were collected, and nitrite levels were quantified by Griess reaction (Sigma-Aldrich) at room temperature for 10 min, using sodium nitrite as standard curve (0-100 *μ*M). Absorbance was measured in a microplate reader (BioTek Instruments, Winooski, VT, USA) at 540 nm.

### 2.13. Survival Assay of Intracellular Amastigotes

To analyze intracellular survival of amastigotes within BMMs in the presence of exosomes, iBMMs were stimulated with aExo in suspension (2.5, 5, 10, 25, and 50 *μ*g/mL) during 24 h in RPMI-1640, supplemented with 10% FBS/-exo at 37°C in 5% CO_2_. As controls, nonstimulated iBMMs and iBMMs stimulated with LPS (100 ng/mL), both in the absence of exosomes, were used. After 24 h, stimulated iBMMs were washed with PBS and further incubated in complete RPMI-1640 medium supplemented with 10% FBS/-exo at 26°C for 72 h, leading to the gradual release of viable amastigotes, which transformed into promastigotes. Differentiated promastigotes were fixed in 2% glutaraldehyde and counted in a Neubauer chamber.

### 2.14. Flow Cytometry

The analysis of antigen presentation and costimulation molecules of infected and stimulated BMMs was analyzed by flow cytometry. BMMs were infected and stimulated, as described above. To detach adherent cells after stimulation, ice-cold PBS was added to each well and iBMMs were released by mechanical pressure. Cells were incubated with anti-CD16/CD32 (Biolegend) for 20 min at 4°C to prevent nonspecific binding. The antigen-presenting molecules were analyzed by staining the cells with mouse mAbs (Biolegend) anti-MHC-I (PE), anti-MHC-II (APC-Cy7), and anti-CD1d (FITC). Costimulatory and coinhibitory molecules were stained with mouse anti-CD86 (PerCP-Cy5.5) and anti-PD-L1 (PE-Cy7), respectively. All the antibodies were diluted 1 : 100 and incubated for 30 min in the dark at 4°C. Cells were then washed with PBS, fixed in 2% paraformaldehyde for 20 min at 4°C and resuspended in PBS for FACS analysis. Events were recorded for individual samples using a FACSCanto II Flow cytometer. Data were analyzed by the FlowJo v.10 software (Treestar, Ashland, OR, USA).

### 2.15. Statistical Analysis

The statistical analyses were performed using the GraphPad Prism v.6 software (GraphPad Software, Inc., CA, USA). Differences between groups were determined by nonparametric Mann–Whitney *U* tests. A value of *p* < 0.05 was considered statistically significant.

## 3. Results

### 3.1. FBS-Derived Exosomes Are Not Required for *L. mexicana* Amastigote Growth

**Figure 1:**
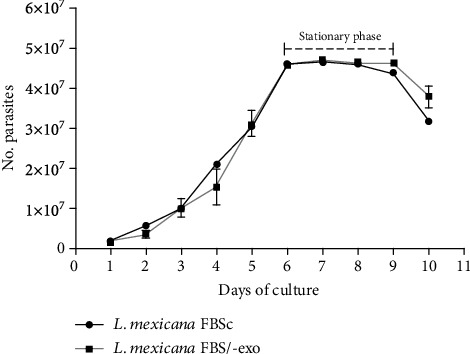
Exosomes derived from FBS are not required for growth of *L. mexicana* amastigotes. Parasite growth was analyzed in RPMI-1640 medium supplemented with normal FBS (control) or FBS/-exo for ten days. Data represent the mean of parasite number ± SD (*n* = 3).

To determine if FBS-derived exosomes are required for parasite growth, kinetic growth curves of *L. mexicana* amastigotes were assessed in Grace's insect medium supplemented with either normal FBS or FBS/-exo. Growth curves showed that amastigotes grew equally well in medium supplemented with FBS/-exo, as compared to normal FBS. A logarithmic growth was observed between days 1 and 6, reaching the stationary phase at day 6 until day 8, after which the parasite culture declined (Figure 1). These results show that exosomes naturally present in FBS are not required for amastigote growth.

### 3.2. Exosomes Secreted by *L. mexicana* Amastigotes Contain gp63 and PP2C

**Figure 2:**
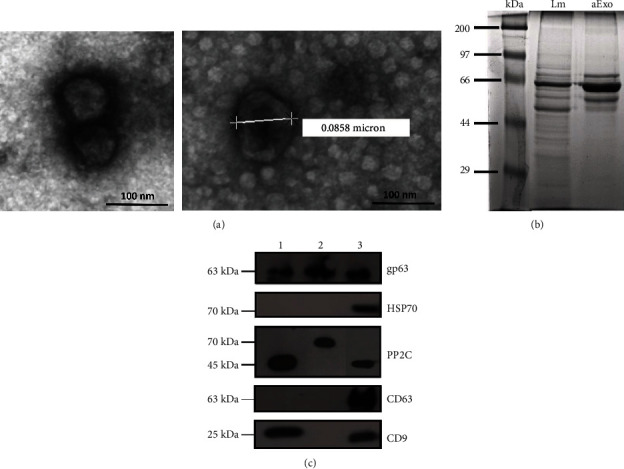
Exosomes released by *L. mexicana* amastigotes contain gp63 and PP2C. Isolated exosomes from supernatants of *L. mexicana* amastigote cultures were visualized by TEM and their protein profile was analyzed. (a) Representative TEM micrographs of aExo with negative staining. Left image shows exosomes contained within membranes (120,000x magnification). Right image exhibits aExo with a diameter of ~85 nm (160,000x magnification). (b) Protein profile of *L. mexicana* amastigotes lysate (Lm) and of their exosomes (aExo) in 10% SDS-PAGE with silver stain. (c) Immunodetection of gp63, HSP70, PP2C, CD63, and CD9 in lysates of *L. mexicana* amastigotes and their secreted exosomes. Lane 1: amastigote lysates; Lane 2: exosome lysates; Lane 3: positive controls. The images are representative of two 2 independent experiments.

To ascertain whether vesicles isolated from the *L. mexicana* amastigote cultures were exosomes, their size and the expression of mammalian exosome markers (CD63 and CD9), as well as the expression of virulence factors (gp63 and PP2C), were evaluated. TEM revealed that the diameter of the isolated vesicles ranged between 80 and 150 nm (Figure 2(a)), demonstrating that isolated vesicles were within the size range of exosomes. The electrophoretic (SDS-PAGE) protein analysis done with 10% silver staining revealed that protein profile in lysates of *L. mexicana* amastigotes differed from the profile of their purified exosomes (Figure 2(b)). Furthermore, Western blotting showed that exosomes isolated from supernatants of *L. mexicana* amastigote cultures did not express the mammalian exosome markers CD63 and CD9 (63 kDa and 25 kDa, respectively) nor HSP70 (70 kDa), although they did express the parasite virulence factors gp63 and PP2C (63 kDa and 70 kDa, respectively). This differed from amastigote lysates, which also showed gp63 and PP2C (45 kDa) expression, in addition to the mammalian exosome marker CD9 (25 kDa) (Figure 2(c)). Taken together, these observations confirm that the microvesicles isolated from *L. mexicana* amastigote cultures were exosomes, since their size was below 150 nm (exosome micrographs are shown in Supplementary 2) and they expressed parasite virulence factors such as gp63 and PP2C, capable of interfering with the host immune response.

### 3.3. Exosomes of *L. mexicana* Are Internalized by BMMs and Colocalize with CD63

**Figure 3:**
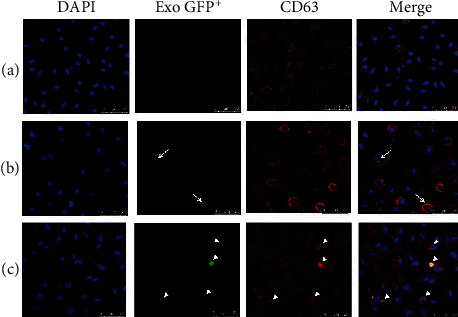
*L. mexicana* exosomes internalized by BMMs colocalize with CD63. Confocal microscopy of BMMs incubated with green fluorescent exosomes for 2 or 6 h. (a) Nonstimulated BMMs. (b) BMMs after 2 h of incubation with exosomes (10 *μ*g/mL). Arrows show green exosomes localized in the cytoplasm of BMMs, and CD63 marker is evidenced on the cell membrane. (c) BMMs incubated for 6 h with exosomes (10 *μ*g/mL). Merged image shows yellow staining (arrowheads) as a result of colocalization of CD63 marker and *L. mexicana* exosomes. DAPI stain shows nuclei in blue, tetraspanin CD63 is stained in red, and exosomes show green staining. The images are representative of two 2 independent experiments.

To demonstrate that exosomes of *L. mexicana* are taken up by macrophages, exosomes were purified from *L. mexicana* parasites transfected with sfGFP (Supplementary 3). BMMs were coincubated with green fluorescent exosomes (10 *μ*g/mL) for 2 or 6 h and analyzed by confocal microscopy. Microphotographs revealed that nonstimulated BMMs did not exhibit green fluorescence in their cytoplasm, although these cells showed the tetraspanin marker CD63 on their cellular membrane (Figure 3(a)). After 2 h of incubation, green exosomes begin to be evidenced in the cytoplasm of BMMs (Figure 3(b)), and after 6 h, exosome internalization increased and some exosomes were found in the perinuclear region, forming aggregates that colocalized with the tetraspanin CD63 marker, possibly in late endosomes (Figure 3(c)). This was not evidenced after 2 h of incubation. These observations demonstrate that *L. mexicana* exosomes were efficiently internalized into macrophages.

### 3.4. aExo from *L. mexicana* Decrease NO Production and Enhance Parasite Survival within iBMMs

**Figure 4:**
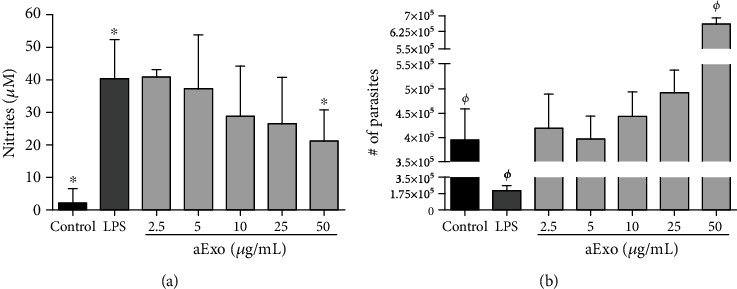
aExo from *L. mexicana* reduce NO production and favor parasite survival within iBMMs. For NO production analysis, iBMMs were coincubated with LPS (100 ng/mL) and aExo in suspension (2.5, 5, 10, 25, and 50 *μ*g/mL) for 24 h, and cell supernatants were collected, and nitrates were quantified. For intracellular survival assay, iBMMs were stimulated with aExo for 24 h, washed, and further cultured for 72 h in RPMI medium supplemented with 10% FBS/-exo at 26°C. Differentiated promastigotes were counted. (a) Nitrites production by iBMMs stimulated with aExo. (b) Survival of intracellular parasites released by BMMs after aExo stimulation. Data are presented as mean ± SD (*n* = 3). Statistically significant differences were determined using Mann–Whitney *U* test; *p* < 0.05 was considered significant, compared to control group.

Given that the *L. mexicana* aExo contain virulence factors, such as gp63 and PP2C that may impair macrophage microbicidal mechanisms, NO production was evaluated in culture supernatants of iBMMs stimulated with LPS (100 ng/mL) and aExo during 24 h. Nonstimulated iBMMs (control) showed no production of nitrates, whereas iBMMs stimulated only with LPS exhibited high levels of nitrite production (40.6 ± 11.5). iBMMs stimulated concomitantly with LPS and aExo showed that exosomes exerted a reduction in nitrite production, which correlated with the concentration of exosomes in a dose-dependent manner. Thus, the most elevated concentration of aExo (50 *μ*g/mL) elicited the most pronounced decrease of nitrite levels (21.4 ± 9.2), as compared with the LPS positive control (Figure 4(a)). To determine whether decreasing levels of NO production induced by aExo stimulation impaired the killing capacity of macrophages, parasite intracellular survival was assessed after 24 h of aExo stimulation. iBMMs stimulated only with LPS (100 ng/mL) showed significantly reduced parasite survival (2.1 × 10^5^), as compared to nonstimulated iBMMs (3.9 × 10^5^). Yet, after incubation with exosomes, an enhanced parasite survival was observed in a dose-dependent fashion, surpassing the number of parasites that survived in the control. Thus, 50 *μ*g/mL of aExo led to a significant increase (6.6 × 10^5^) of viable parasite release of iBMMs (Figure 4(b)). Taken together, these data provide evidence that aExo of *L. mexicana* downregulate macrophage microbicidal activity exerted by NO production, leading to an increase of intracellular parasite survival.

### 3.5. *L. mexicana* aExo Modulate MHC-I and CD1d Expression on iBMMs

**Figure 5:**
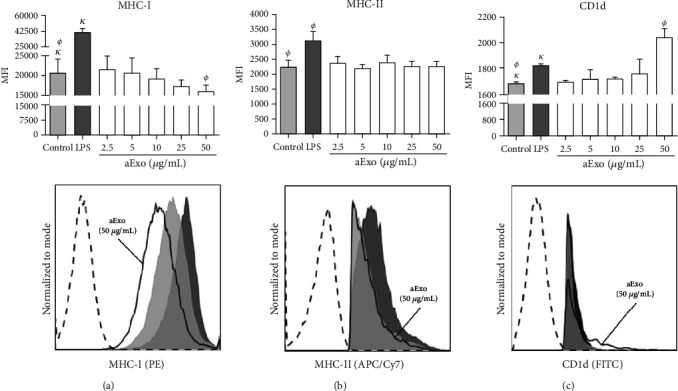
*L. mexicana* aExo modulate MHC-I and CD1d expression on iBMMs. Infected BMMs were incubated with different concentrations of aExo (2.5, 5, 10, 25, and 50 *μ*g/mL) for 24 h. Cells were stained with mAbs anti-MHC-I, anti-MHC-II, and CD1d. (a) MHC-I expression. (b) MCH-II expression. (c) CD1d expression. Nonstimulated iBMMs and positive control of iBMMs incubated with LPS are presented. Top panels exhibit statistical summary bar graphs of mean fluorescence intensity (MFI) ± SD (*n* = 5). Statistically significant differences were determined using Mann–Whitney *U* test; *p* < 0.05 was considered significant, compared to control group. Bottom panels show representative flow cytometry histograms of molecule expressions. Nonstimulated iBMMs (gray filled histograms), positive control (black filled histograms), and iBMMs stimulated with 50 *μ*g/mL of aExo (black line) are shown. Unstained control is shown by a black dashed line.

To evaluate whether aExo of *L. mexicana* modulate the expression of antigen-presenting molecules, expression of MHC and CD1d were analyzed on iBMMs stimulated with exosomes for 24 h. A progressive decrease on the expression of MHC-I was observed, which became significant at exosome concentrations above 50 *μ*g/mL, where a 22.6% reduction of MHC-I expression was evidenced, as compared to nonstimulated iBMMs (20,742 ± 3,384 for control vs. 16,048 ± 1,668 for aExo 50 *μ*g/mL) (Figure 5(a)). No modification was observed on MHC-II expression after aExo stimulus for 24 h (Figure 5(b)). In contrast, CD1d expression increased 18.2% after incubation with aExo 50 *μ*g/mL, the highest concentration tested (1,681 ± 10.6 for control vs. 1,987 ± 124.2 for aExo 50 *μ*g/mL) (Figure 5(c)). The positive control of iBMMs stimulated with LPS (in the absence of exosomes) showed enhanced expression of all antigen-presenting molecules, as compared to nonstimulated iBMMs (*p* < 0.05). These data suggest that aExo modulated antigen-presenting molecules on infected macrophages.

### 3.6. *L. mexicana* aExo Downregulate Costimulatory Molecules on iBMMs

**Figure 6:**
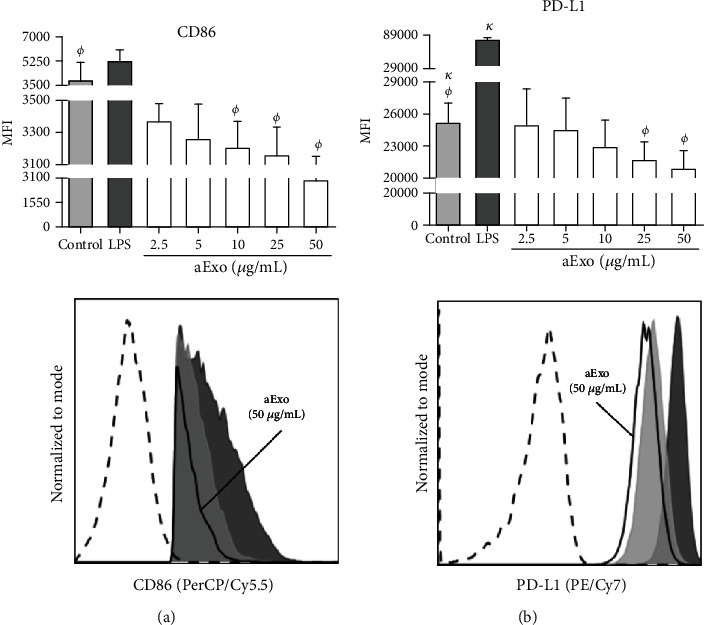
aExo of *L. mexicana* downregulate CD86 and PD-L1 expressions on iBMMs. Infected BMMs were incubated with aExo (2.5, 5, 10, 25, and 50 *μ*g/mL) for 24 h. Cells were stained with mAbs anti-CD86 and anti-PD-L1. Controls: nonstimulated control and control of iBMMs incubated with LPS. (a) CD86 expression. (b) PD-L1 expression. Top panel displays a bar graph of MFI ± SD (*n* = 5). Statistically significant differences were determined using Mann–Whitney *U* test; *p* < 0.05 was considered significant, compared to control group. Bottom panels show representative histograms of molecule expressions. Nonstimulated iBMMs (gray filled histograms), positive control (black filled histograms), and iBMMs stimulated with 50 *μ*g/mL of aExo (black line) are shown. Unstained control is shown in black dashed line.

Considering that aExo from *L. mexicana* altered the expression of antigen-presenting molecules on iBMMs, the expressions of costimulatory molecules were also analyzed on iBMMs stimulated with amastigotes exosomes. These included the activation molecule CD86 and the inhibitory costimulatory molecule PD-L1. A significant dose-dependent reduction was observed on the expression of both molecules on iBMMs after incubation of aExo concentrations above 10 *μ*g/mL (*p* < 0.05) (Figure 6). The decrease of CD86 expression was approximately 60% (4,694 ± 1,473 of control vs. 2,996 ± 157.9 for aExo 50 *μ*g/mL) (Figure 6(a)), whereas the reduction of PD-L1 was around of 20% on stimulated cells with 50 *μ*g/mL of aExo (25,192 ± 1,793 of control vs. 20,917 ± 1,707 for aExo 50 *μ*g/mL) (Figure 6(b)), as compared to the nonstimulated iBMMs. The positive control of iBMMs stimulated with LPS led to the enhanced expression of PD-L1, compared to control (*p* < 0.05), but no significant change was observed on the CD86 expression. Taken together, these observations suggest that aExo of *L. mexicana* downregulate costimulatory molecules, such as CD86 and PD-L1.

## 4. Discussion

Exosomes are endocytic membrane-derived nanovesicles that contain functional biomolecules including DNA, RNA, mRNA, miRNA, lipids and proteins. They travel in bodily fluids and are capable of modifying cellular responses, such as the immune response [16, 30]. They are internalized by endocytosis or membrane fusions, and once inside of cells, such as macrophages, they are transported in endosomes and colocalize with tetraspanin CD63 in late endosomes [31]. In addition to CD63 marker, exosomal membranes are enriched in other endosomal-specific tetraspanins such as CD9, CD81, CD82 and CD37 [32]. *Leishmania* exosomes are loaded with membrane and cytoplasmic proteins in addition to virulence factors of the parasite [33]. Here, we analyzed exosomes of *L. mexicana* amastigotes and evaluated their effect on parasite-infected BMMs. We found that they not only inhibit nitric oxide production, favoring parasite survival within the cells, but they also modulate antigen-presenting molecules in the infected BMMs.

To avoid possible interference by FBS exosomes in our study, these were eliminated prior to all experiments. This was important since FBS exosomes are able to induce phenotypic changes and affect cellular proliferation and migration, as shown in tumor cells [24, 34–36]. Interestingly, depletion of FBS exosomes had no effect on the growth of *Leishmania* amastigotes, and it could be hypothesized that FBS exosomes possibly lack specific receptors or homologous signaling pathways related to proliferative responses of the parasite.

Exosomes of *L. mexicana* amastigotes have an average diameter of approximately 85 nm, which is within the limits (30-150 nm) described for exosomes of eukaryotic cells [15]. The protein analysis of amastigote exosomes showed that its content differed from that of amastigote lysates, which is in accordance with the literature, where more proteins have been reported in parasite extracts as compared to their exosomes [20]. Exosomal markers, such as tetraspanins CD63, CD9, or HSP70, were not found in amastigote exosomes. This observation also proved indirect evidence that no contamination with FBS exosomes was present in our study. Although the absence of tetraspanins CD63 and CD9 was to be expected, the lack of HSP70 in amastigote exosomes was noteworthy, since this protein had previously been described in *Leishmania* promastigotes, subjected to heat-stress [33]. We hypothesize that the absence of HSP70 in our study might be explained by the fact that *L. mexicana* amastigotes were cultured at 33°C, during which the parasites are not exposed to thermal stress conditions and therefore do not express HSP70.

Virulence factors contained within *Leishmania* exosomes are capable of interacting with host phosphatases, including gp63, aldolase (fructuose-1-6-biphosphate aldolase), and EF-1*α*. These are released in the parasitophorous vacuoles and reach the cytosol, as has been shown for macrophages infected with *L. donovani* [21, 33]. We now demonstrate that exosomes of *L. mexicana* amastigotes contain the metalloproteinase gp63 and the phosphatase PP2C, proteins that have been reported to be secreted by *Leishmania* [37, 38]. The zinc-metalloprotease gp63 plays a prominent role in establishing conditions for the differentiation of promastigotes to amastigotes, being differentially expressed in *Leishmania* sp. Gp63 is predominantly expressed in promastigotes and downmodulated in the amastigote stage in *L. major*. However, it has been reported as the most abundant protein on amastigotes in *L. mexicana* and downmodulated in the promastigote differentiation stage [39, 40]. This major surface glycoconjugate secreted by the parasite interferes with many host defense mechanisms, including the cleavage of components of signaling cascades of fusion molecules and hampering the phagolysosomal fusion [8]. It has been shown to localize in the nuclear envelope and perinuclear area of host cells [41]. Gp63 cleavage of host cell substrates can affect transcription factors c-Jun and NF-*κ*B (p65/RelA fragment), as well as host phosphatases, including SHP-1 (Src homology region 2-domain-containing phosphatase-1) [7, 41, 42]. The proteolytic activation of host phosphatases, such as SHP-1, can lead to inhibition of signaling pathways in the host cells required for parasite control [7]. Thus, by subverting multiple microbicidal and immune functions of the cell, gp63 helps create a safe niche for parasite replication.

In addition to modulating host phosphatases, parasite virulence factors contained in exosomes are the parasite's own phosphatases that can interfere with phosphorylation events in host cells. Our data now show that exosomes of *L. mexicana* amastigotes contain the phosphatase PP2C, a metal-dependent protein phosphatase that dephosphorylates serine/threonine substrates, thereby negatively regulating protein kinase cascades [43]. This enzyme has been proposed to regulate key cellular events by downregulating MAPK signaling pathway, thereby also affecting the oxidative burst and iNOS production [44, 45]. In leishmaniasis, it is regarded a virulence factor and has been reported in vesicles and in the flagellar pocket of *Leishmania* amastigotes and promastigotes, where exosome secretion takes place [37]. The PP2C phosphatase of *Leishmania major* was shown to have a molecular mass of 44.9 kDa [37]. Antibodies produced against *L. major* PP2C recognized a ~70 kDa protein in the secretome fraction of the *L. mexicana* parasite, possibly corresponding to a glycosylated form of the protein [10]. This same antibody was used in our current study, and it is noteworthy that this antibody recognizes a ~70 kDa protein in exosomes of *L. mexicana* amastigotes, which has the same molecular weight of PP2C in the secretome fraction, where exosomes are also present. Another explanation for the weight of PP2C could be that a protein homologous to PP2C could be present in exosomes of *L. mexicana* amastigotes. This is plausible, since 15 different PP2C proteins with similar sequences have been described by a phylogenetic analysis in *L. major* [46]. Other phosphatases found in exosomes of different *Leishmania* species that possibly play a role as virulence factors include LmPRL-1, which are predominantly expressed and secreted by exosomes of *L. major*, evidenced during macrophage infections. This parasite phosphatase was also found in the cytoplasm of the host cells and suspected to modulate signaling pathways by targeting host cell phosphoproteins, thereby interfering with cellular functions and contributing to the survival of the parasite [21]. It would be of interest to study their function also in *L. mexicana* infection.

The use of green exosomes obtained from sfGFP transfected *L. mexicana* parasites showed that they appear in the cytosol of BMMs after 2 h of incubation and increased throughout the next 6 h. Exosomes can enter cells through diverse routes, such as phagocytosis, receptor-mediated endocytosis, or by membrane fusion [17, 47]. In mammalian exosomes, an internalization motif based on tyrosine has been described in the C-terminal of the CD63 tetraspanin, which leads to their rapid endocytosis [48]. Yet, since *L. mexicana* exosomes do not express CD63, another unknown pathway of entry seems to be present. A proteomic analysis has shown that *L. major* exosomes contain orthologous proteins to the clathrin heavy chain and target SNARE proteins found in the mammalian exosome proteome, which are central components of endocytosis and eukaryotic fusion mechanisms [33, 49]. This possibly suggests that exosomes enter cells through clathrin-mediated endocytosis or SNARE protein-mediated membrane fusion [49]. Once inside BMMs, *L. mexicana* exosomes were found to colocalize with the tetraspanin CD63 (present in late endosomes and lysosomes) after 6 h. This is suggestive of a possible fusion between parasite and macrophage exosomes and that possibly parasite exosomes are recycled in macrophage endosomes [50]. After their uptake by phagocytic cells, *Leishmania* amastigotes release proteins and exosomes in parasitophorous vacuoles, a phagolysosome-like structure that shelters the intracellular amastigotes [51]. These later which fuse with multiple structures including late endosomes, also known as MVBs [18, 51]. Thus, macrophages possibly eliminate parasite proteins through their exosomes that derive from late endosomes [13]. By systematically delivering messages through exosomes, parasite amastigotes are able to modulate the response of their host cells in addition to becoming recirculated to other cells, favoring disease progression [13]. This is in accordance with a report on patients with tuberculosis, where exosomes containing *Mycobacterium tuberculosis* proteins were found in serum of the patients, possibly contributing to the infection [52].

Our data now show that exosomes of *L. mexicana* amastigotes, in addition to inhibiting NO production of macrophages, they also exert an impact on host immune mechanisms by modifying antigen-presenting and costimulatory molecules, both of which have a crucial role in the immune defense against the parasite [53]. Hampering leishmanicidal mechanisms of macrophages through reduction of the NO production, in combination with reducing antigen-presenting mechanisms, limits the functional capacity of these cells to activate the adaptive immune response [53]. Our report is in accordance with previous reports that have shown that *L. mexicana* promastigotes, as well as their exosomes, inhibit NO production in the macrophage cell line B10R [54]. Furthermore, *L. major* exosomes were shown to downregulate the *NOS2* gene and degrade NF-*κ*B and AP-1 subunits, possibly by their gp63 [20]. Based on these observations, we now speculate that the inhibition of NO and the downregulation of MHC-I, CD86 and PDL-1 expressions in iBMMs coincubated with exosomes of *L. mexicana* amastigotes are possibly linked to degradation of NF-*κ*B by exosome gp63. It is noteworthy that the MHC-II expression remained unchanged, possibly because this molecule forms part of a protein complex related to multiple transcription factors, the most important of which is the class II transactivator (CIITA), the master regulator of class II gene activation, and the protein expression is regulated by transcription factors STAT-1*α*, IRF-1 and USF-1 [55]. This result has also been observed for *L. infantum* parasites and their exosomes, which did not affect the expression of MHC-II in BMDCs and BMMs [22]. It now remains to be established, whether the reduced expression of MHC-I and costimulatory molecules induced by amastigote exosomes affects CD8 T cell activation in *Leishmania* infections.

It is noteworthy that our data on enhanced CD1d expression caused by *L. mexicana* aExo in iBMMs contrasts with reports in the literature, where exosomes of *L. major* promastigotes have been reported to reduce mRNA for CD1d in the cell line B10R [20]. Since the expression of CD1d is controlled by the transcription factor C/EBP-*β* [56], it remains to be established whether this opposing effect can be related to differences in parasite species and/or to the fact that exosomes of different parasite developmental stages were used. The enhanced expression of CD1d possibly suggests that amastigote exosomes could facilitate glycolipid antigen presentations, such as ceramides and sphingolipids of exosome surfaces or parasite glycolipids, such as lipophosphoglycan (LPG), glycoinositol phospholipids (GIPLs), glycosphingophospholipid (GSPL) and glycosylphosfatidylinositol (GPI) [57, 58]. These could be presented to NKT cells by CD1d, thereby inducing cytokine production [58]. However, since this enhanced CD1d expression occurs concomitantly with a reduction of the costimulatory molecule CD86, this possibly suggests that cellular activation is limited.

It was noteworthy that the expression of PD-L1 was found to be reduced on *L. mexicana*-infected BMMs stimulated with aExo. The contrary effect was expected, since in leishmaniasis an inhibition of T lymphocytes has been reported [59]. Moreover, the reduction of PD-L1 became more pronounced after incubating the iBMMs with higher levels of exosomes. Since the transcription of PD-L1 is also regulated by NF-*κ*B [60], a possible explanation for its reduction could be the before-mentioned degradation of NF-*κ*B by exosomal gp63.

Our data now enrich knowledge on the pathogenic mechanisms exerted by amastigote exosomes, aiding intracellular parasite survival. Thus, *Leishmania* amastigotes secrete exosomes inside of parasitophorous vacuoles [51], which lead to a reduction of the free radical NO, avoiding the hostile microenvironment within the cell. We propose that exosomes then fuse with proteins of the endosome membrane of the infected macrophages and are released into the cytosol of the host cells. While being in the perinuclear space [41], parasite exosomes are able to modulate microbicidal mechanisms of infected macrophages by phosphatases contained in their exosomes (PP2C) and by modulating protein tyrosine phosphatases of the host cells (SHP-1) through gp63, thereby inhibiting the nuclear translocation of transcription factors, such as NF-*κ*B, JAK/STAT-1 and AP-1 [6]. The inactivation of NF-*κ*B and other transcription factors can lead to a reduced expression of MHC-I and costimulatory molecules CD86 and PD-L1, which hampers antigen presentation by the infected cells. Yet, despite enhancing CD1d expression when present in large numbers, exosomes still impair an effective immune response, since the lack of costimulatory molecules favors tolerance and cellular anergy [61, 62]. The release of exosomes to the extracellular medium through exocytosis appears to be an important pathway that permits the parasite to export its virulence factors outside the host cells. The interplay of these mechanisms generates conditions leading to enhanced parasite survival within the cell, in addition to permitting the release and spread of abundant parasites to neighboring cells.

## 5. Conclusions

Taken together, we now show that exosomes of *L. mexicana* amastigotes measure ~85 nm, containing protein-phosphatase PP2C (not previously described in exosomes) and the protease gp63. Parasite exosomes are internalized by BMMs and colocalize with the tetraspanine CD63 of endosomal membranes. In infected BMMs, exosomes reduce NO production, favoring parasite survival. They negatively modulate the immune response of the host cell by reducing MHC-I and CD86, both of which hamper effective T cell activation.

## Data Availability

The data used to support the findings of this study are included within the article.

## Supplementary Materials

Supplementary Materials Supplementary 1: flow cytometry analysis of FBS-derived exosomes depletion through ultracentrifugation. (a) Flow cytometer was calibrated with reference FITC+ beads (0.1-2 *μ*m). (b) Flow cytometry analysis of complete normal FBS (FBSc). (c) Flow cytometry analysis of exosome-free FBS (FBS/-exo). Supplementary 2: transmission electron microscopy of *L. mexicana* exosomes. A moderate number of vesicles were observed with a negative staining technique, contrasted with 2% uranyl acetate. (a–e) Photomicrographs of vesicles whose diameters range between 30 and 150 nm in diameter corresponding to exosomes. Bar: 100 nm. (b, f, h) In these exosomes, it is possible to appreciate the lipid bilayer membrane of exosomes (white arrows). (f) Various exosomes are observed in the field. Bar: 500 nm. Some exosomes showing darker staining incorporated a larger amount of contrasting agent. Supplementary 3: fluorescence analysis of transfected *L. mexicana* promastigotes with sfGFP gene. Parasites were transfected with sfGFP gene through the pLEXSY expression plasmid as described Diupotex et al. 2019 [27]. (a) Flow cytometry histogram shows wild-type *L. mexicana*^WT^ parasites (unfilled histogram) and *L. mexicana*^sfGFP^ parasites transfected with sfGFP gene (gray filled histogram). (b) Epifluorescence micrograph of *L. mexicana*^sfGFP^ parasites.
