# Supplementary material for: Leishmania mexicana: Novel Insights of Immune Modulation through Amastigote Exosomes
Source: J Immunol Res. 2020 Dec 2;2020:8894549. doi: 10.1155/2020/8894549 (PMC7728480; doi:10.1155/2020/8894549)
Supplement: Supplementary Materials — Supplementary 1: flow cytometry analysis of FBS-derived exosomes depletion through ultracentrifugation. (a) Flow cytometer was calibrated with reference FITC+ beads (0.1-2 μm). (b) Flow cytometry analysis of complete normal FBS (FBSc). (c) Flow cytometry analysis of exosome-free FBS (FBS/-exo). Supplementary 2: transmission electron microscopy of L. mexicana exosomes. A moderate number of vesicles were observed with a negative staining technique, contrasted with 2% uranyl acetate. (a–e) Photomicrographs of vesicles whose diameters range between 30 and 150 nm in diameter corresponding to exosomes. Bar: 100 nm. (b, f, h) In these exosomes, it is possible to appreciate the lipid bilayer membrane of exosomes (white arrows). (f) Various exosomes are observed in the field. Bar: 500 nm. Some exosomes showing darker staining incorporated a larger amount of contrasting agent. Supplementary 3: fluorescence analysis of transfected L. mexicana promastigotes with sfGFP gene. Parasites were transfected with sfGFP gene through the pLEXSY expression plasmid as described Diupotex et al. 2019 [27]. (a) Flow cytometry histogram shows wild-type L. mexicanaWT parasites (unfilled histogram) and L. mexicanasfGFP parasites transfected with sfGFP gene (gray filled histogram). (b) Epifluorescence micrograph of L. mexicanasfGFP parasites. [file 8894549.f1.docx]

**Supplementary Materials**


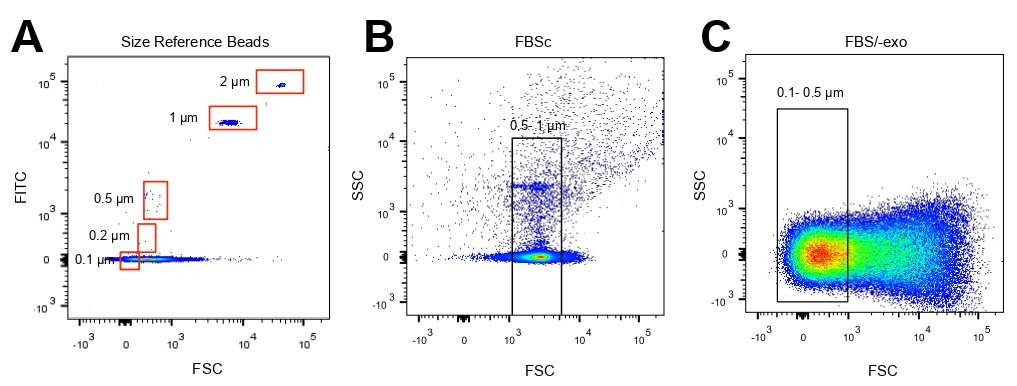


**Supplementary 1.** **Flow cytometry analysis of FBS-derived exosomes depletion through ultracentrifugation**. A) Flow cytometer was calibrated with reference FITC+ beads (0.1-2 μm). B) Flow cytometry analysis of complete normal FBS (FBSc). C) Flow cytometry analysis of exosome-free FBS (FBS/-exo).


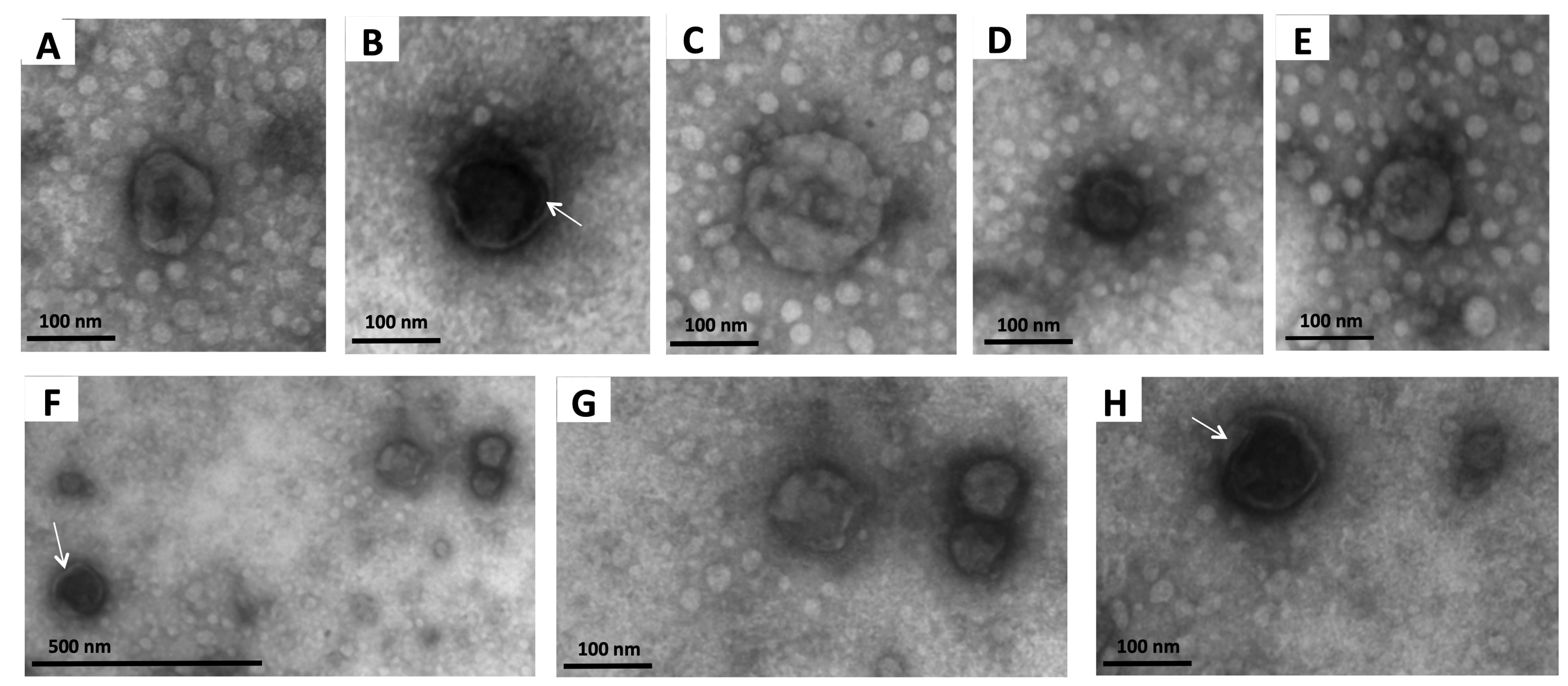


**Supplementary 2. Transmission electron microscopy of *L. mexicana* exosomes.** A moderate number of vesicles was observed with a negative staining technique, contrasted with 2% uranyl acetate. A-E, photomicrographs of vesicles whose diameters range between 30-150 nm in diameter corresponding to exosomes, Bar: 100 nm. B, F and H, in these exosomes it is possible to appreciate the lipid bilayer membrane of exosomes (white arrows). F, various exosomes are observed in the field. Bar: 500 nm. Some exosomes showing darker staining incorporated a larger amount of contrasting agent.


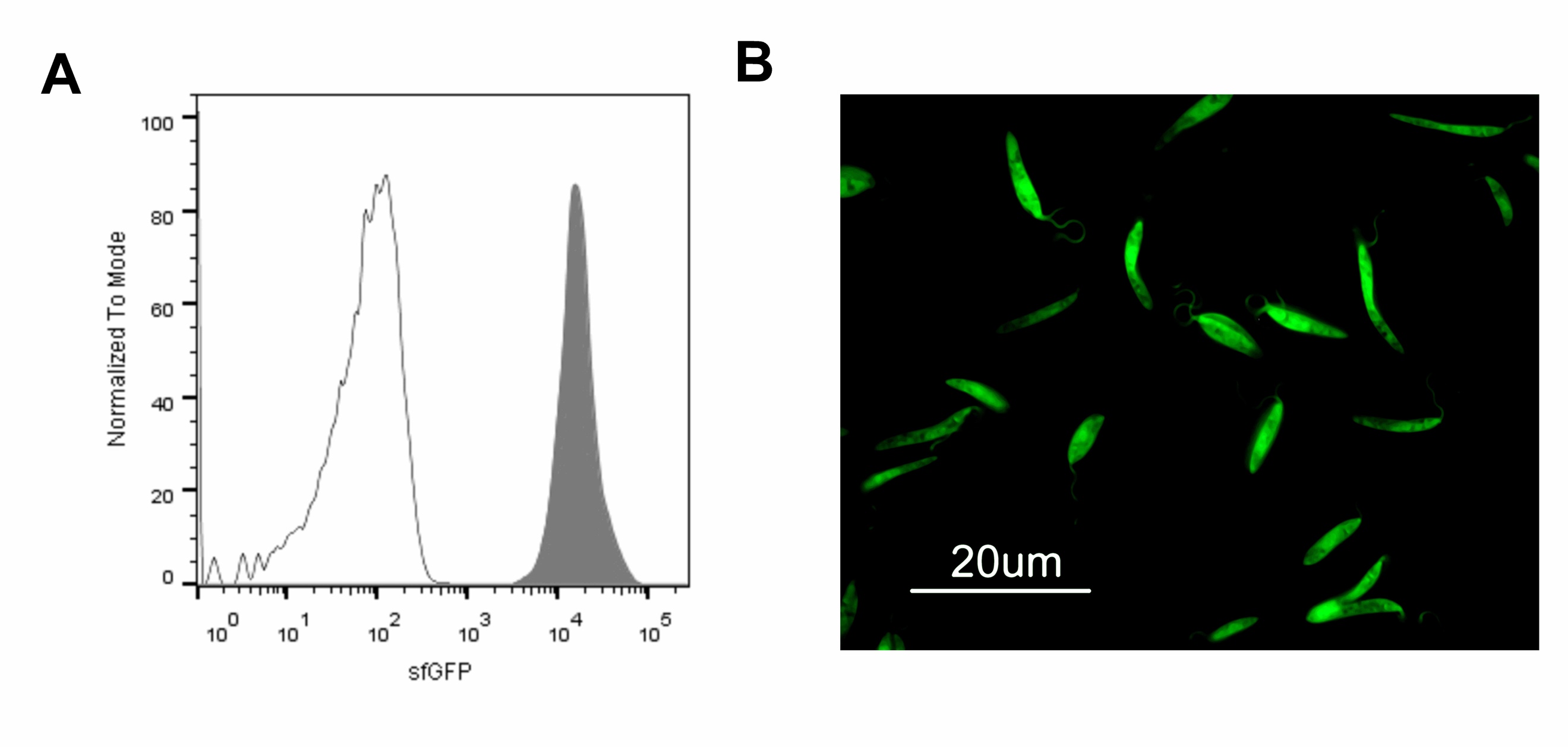


**Supplementary 3. Fluorescence analysis of transfected *L. mexicana* promastigotes with sfGFP gene**. Parasite were transfected with sfGFP gene through the pLEXSY expression plasmid as described Diupotex et al., 2019.^27^ A) Flow cytometry histogram shows wild-type *L. mexicana*^WT^ parasites (unfilled histogram) and *L. mexicana*^sfGFP^ parasites transfected with sfGFP gene (gray filled histogram). B) Epifluorescence micrograph of *L. mexicana*^sfGFP^ parasites.
